# Is computed tomography the ideal method for the identification and management of lung disease in systemic sclerosis?

**DOI:** 10.1590/0100-3984.2016.49.5e2

**Published:** 2016

**Authors:** Agnaldo José Lopes

**Affiliations:** 1 MD, Adjunct Professor of Pulmonology at the Universidade do Estado do Rio de Janeiro (UERJ), Rio de Janeiro, RJ, Brazil. E-mail: agnaldolopes.uerj@gmail.com.

Involvement of the respiratory system is quite common in the course of systemic sclerosis (SSc) and can affect all its components, including the parenchyma, vasculature, airways, pleura, and muscle tissue^(1)^. However, the forms that are the most common and have the greatest clinical repercussions are interstitial lung disease (ILD) and pulmonary arterial hypertension, which account for up to 90% and 60% of cases, respectively^(1,2)^. The presence of a pulmonary lesion is a determining factor in the quality of life of patients with SSc and currently represents the main cause of SScrelated mortality, especially among patients diagnosed with ILD^(1)^. The onset of SSc-ILD is insidious, with subtle clinical symptoms, which explains why the disease is often diagnosed in its advanced stages, when extensive pulmonary fibrosis is already present. Targeted strategies to identify the risk of SSc-ILD at the beginning of its course are fundamental, because such strategies can allow the early introduction of treatment^(3)^.

The importance of differentiating histological patterns in SSc-ILD is that it determines the prognosis, which is better in cases of nonspecific interstitial pneumonia and worse in those of usual interstitial pneumonia^(2)^. However, in clinical practice, conducting routine lung biopsy is not recommended, being reserved for atypical clinical presentations and tomography findings^(2)^. In this issue of **Radiologia Brasileira**, Bastos et al.^(4)^ provide an interesting review in which they emphasize the importance of high-resolution computed tomography (HRCT) and discuss the main tomography patterns of lung disease in SSc. The use of HRCT, in conjunction with pulmonary function tests, plays a critical role in the evaluation of the treatment and prognosis of SSc-ILD^(5)^. The HRCT method is more precise in differentiating between airway abnormalities and changes that occur in the parenchyma, even allowing the quantitative assessment of lung disease^(3,5,6,8)^. It is considered the gold standard in the investigation of SSc-ILD, with a sensitively > 90%, given that the accuracy of clinical and functional assessments is much lower. In fact, more than 60% of cases of SSc-ILD diagnosed by HRCT feature normal pulmonary function test results, indicating that such tests perform poorly in screening for ILD^(3)^.

After all, what is the role of HRCT in the management of lung disease in SSc? Although honeycombing is an indicator of poor prognosis^(2)^, the main marker of unfavorable evolution is the extent of the disease, which, when combined with the deterioration of lung function, best defines the prognosis^(1)^. The absence of findings consistent with fibrosis in the initial HRCT of patients with SSc is highly predictive of a "fibrosis-free" follow-up HRCT, whereas a finding of more than 20% fibrosis in the initial exam is associated with a high annual fibrosis progression rate, a decline in lung function, and the development of pulmonary arterial hypertension^(3)^.

As stated in the study conducted by Bastos et al.^(4)^, there is also growing interest in identifying patients with rapidly progressive disease, because such patients have an increased risk of progression of ILD and therefore require intervention as early as possible. That makes the use of HRCT extremely attractive in the monitoring of SSc-ILD. However, the frequent use of ionizing radiation is a matter of growing concern, especially in this population that already has an increased risk of tumors, as mentioned by Bastos et al.^(4)^. Therefore, new imaging techniques, such as ultrasound of the thorax, the reduction in the number of HRCT slices, and the use of low-dose CT, have been proposed as options to resolve this problem^(9)^.

More recently, MRI has been shown to be a technique with potential to detect and classify SSc-ILD, showing good correlation with HRCT (*r* = 0.85; *p* < 0.001). However, the use of MRI is still limited to certain cases in which there is a need to minimize the use of ionizing radiation^(9,10)^. Allied to this problem, the upcoming addition of new antifibrotic drugs, such as pirfenidone^(11)^, to the therapeutic arsenal against SSc will further increase the challenges facing radiologists in the search for an ideal method to monitor patients with the disease. After all, to live is to face challenges, and the art of practicing medicine brings new challenges every day!
